# Salmagundi

**Published:** 1884-10

**Authors:** 


### SALMAGUNDI.

EDITED BY E. G. BETTY, D.D.S.

### ECHOES OF THE 24TH MEETING OF THE A. D. A.

*The Cosmos*, *New England Journal*, *Archives of Dentistry*, and the REGISTER, all had reporters on hand making reports. That's enterprise for you, and shows that the profession is bound to have them willy nilly and not wait for the tardy transactions.

OUR good friend, Dr. Frederichs, of New Orleans, crossed the continent at almost its long diameter to attend the A. D. A., whose meetings he rarely misses. The Doctor is looking very well and feeling younger the older he grows. May his face long be seen amongst us.

WE are sorry to record that Dr. Jerry Robinson (may his foil increase) announced incidentally that this was in all probability the last meeting of the A. D. A. that he should ever attend. This will be regretted by hosts of friends, and more especially by those who were conspicuous mainly by their absence.

A VERY amusing feature of the discussion on Herbst's method of filling was, that no one having remarks to make on the subject had any knowledge of it, as each one prefaced his speech with an apology for his second-hand information and his failure of success because he could not get the kind of gold that Dr. Herbst uses. Selah!

---

THE 22nd annual announcement of the Philadelphia Dental College and of the Hospital of Oral Surgery is received, giving evidence of increased prosperity.

---

A ST. LOUIS man twenty-five years old, is the victim of a queer mania. He firmly believes that he is seventy years old. There are some equally queer cases in Philadelphia, the victims being women who are seventy years old, yet firmly believe they are twenty-five.—[*Philadelphia Call.*

The exciting causes of dental caries is still an open question, as also is the character of the material entering into the composition of the North Pole. Both of these things are great *bug-bears* anyhow.

---

The young Chicago Dental College has started out in the right way by sending representatives to the meeting at New York and taking a prominent part in the proceedings of the newly organized College Faculties Association.

---

### A LIVELY OLD GIRL.

Mrs. Nancy Skidmore, of Manhattan Island, claims to be 120 years old. "Do you ever use liquor?" asked some recent visitors. "Ever since I was seventeen years old. I drink about a quart of strong whisky every day, and Dr. Blagdon, of Harlem, says it's the only thing that keeps me alive. Yes, I have smoked ever since I was twenty-three, sometimes a pound of tobacco a day. Here's a pipe I've used for fifty-five years." The lively old lady accompanied her visitors to the door, and as they were departing cried: "The girls didn't eat ice cream or wear Mother Hubbards when I was a girl," and then she threw a brick at the goat, and re-entered the house.—Daily Paper.

---

A French scientific person has investigated the consciousness of the decapitated head, and has found that it remains in possession of all the faculties if the hemorrhage does not pass certain limits, and if the proportion of oxygen in the blood is sufficient for keeping up the nervous functions, but that this can never exceed half a minute of time. But this is time enough for a great deal of thinking. To know the thoughts at that stage is interesting. A notion runs that as the mind gets to the border it has new light behind and before. The testimony of the feeble-minded and fearful moribund is thought more of than that of the strength of their lives. What the cut off head thinks would be a great addition to these testimonies. Since mind-reading and instantaneous photographing, the taking of the off head's reflections and anticipations may not seem an impossibility. S. R. R.

### VON BONHORST'S ANÆSTHETIC.

Though we don't know what are the ingredients of this preparation, yet we do know that it is in its way a good thing. A short time ago the writer was called to extract a tooth at the residence of a lady patient. As she had suffered greatly for several days with acute periostitis, and being very timid, it was desirable to obtund the pain of the extraction as much as possible. So the anæsthetic was hit upon. The sponges of the applicator were saturated and applied to the gums around the tooth (a lower left bicuspid), and held there for several minutes. The gums were also bathed with the anæsthetic. The result was that the pain disappeared. The lancing was not felt; neither was the adjustment of the forcep. The *pull* produced the usual pain, but it was quickly over. The gums were again bathed with the anæsthetic, and in a short time the pain from the shock was considerably diminished. If the medicament can modify the pain as much as this in an extreme case, it certainly is a good thing to have about the office, as, in addition, it may be used for other purposes than extraction.

---

### QUERIES.

Will Miss Sal Magundi be kind enough to answer a couple of questions?

1st.—Is it good usage to employ two titles which are synonymous, or when one is embraced by the other? I ask this because I have upon several occasions seen it used by no less an authority than the REGISTER. For instance, *Dr.* James Taylor, *M.D.*, *D.D.S.*

2d.—When a physician or dentist takes up his residence in a new community, is it customary for his professional brethren *first* to call on him, or for the new comer first to call upon the resident practitioners? Respectfully, Q. E. X.

1st.—It is not generally the custom to prefix the "Dr." and then add the initials denoting a degree or degrees, and when you see both in print as you instance, it can be put down as an oversight or a slip of the pen. The simple term "Doctor" is usually the one used, though it has a numerous significance. For all professional purposes the titles following the name might, in order to save time, space, and printer's ink, be condensed thus, "M.D., etc.," thereby saving the individual the charge of egotism, for it is certainly abominable to see a name followed by half a galley of descriptive titles, the list usually winding up with the welcome "etc.," anyhow.

2d.—In social life the neighbors make the first call, and the custom might with propriety apply to gentlemen in their professional capacity, though the courtesy is "more honored in the breach than the observance." The societies are the usual media through which practitioners become acquainted with one another.

